# A Comparison of Y-Chromosome Variation in Sardinia and Anatolia Is More Consistent with Cultural Rather than Demic Diffusion of Agriculture

**DOI:** 10.1371/journal.pone.0010419

**Published:** 2010-04-29

**Authors:** Laura Morelli, Daniela Contu, Federico Santoni, Michael B. Whalen, Paolo Francalacci, Francesco Cucca

**Affiliations:** 1 Dipartimento di Zoologia e Genetica evoluzionistica, Università di Sassari, Sassari, Italy; 2 Dipartimento di Scienze Biomediche, Università di Sassari, Sassari, Italy; 3 Laboratorio di Immunogenetica, Ospedale Microcitemico, Cagliari, Italy; 4 Biomedical Application Area, Center for Advanced Studies, Research and Development in Sardinia (CRS4), Pula, Italy; 5 Swiss Institution for Bioinformatics, University of Geneva, Geneva, Switzerland; 6 Istituto di Neurogenetica e Neurofarmacologia (NN-CNR), Monserrato, Italy; Institute of Evolutionary Biology (CSIC-UPF), Spain

## Abstract

Two alternative models have been proposed to explain the spread of agriculture in Europe during the Neolithic period. The demic diffusion model postulates the spreading of farmers from the Middle East along a Southeast to Northeast axis. Conversely, the cultural diffusion model assumes transmission of agricultural techniques without substantial movements of people. Support for the demic model derives largely from the observation of frequency gradients among some genetic variants, in particular haplogroups defined by single nucleotide polymorphisms (SNPs) in the Y-chromosome. A recent network analysis of the R-M269 Y chromosome lineage has purportedly corroborated Neolithic expansion from Anatolia, the site of diffusion of agriculture. However, the data are still controversial and the analyses so far performed are prone to a number of biases. In the present study we show that the addition of a single marker, DYSA7.2, dramatically changes the shape of the R-M269 network into a topology showing a clear Western-Eastern dichotomy not consistent with a radial diffusion of people from the Middle East. We have also assessed other Y-chromosome haplogroups proposed to be markers of the Neolithic diffusion of farmers and compared their intra-lineage variation—defined by short tandem repeats (STRs)—in Anatolia and in Sardinia, the only Western population where these lineages are present at appreciable frequencies and where there is substantial archaeological and genetic evidence of pre-Neolithic human occupation. The data indicate that Sardinia does not contain a subset of the variability present in Anatolia and that the shared variability between these populations is best explained by an earlier, pre-Neolithic dispersal of haplogroups from a common ancestral gene pool. Overall, these results are consistent with the cultural diffusion and do not support the demic model of agriculture diffusion.

## Introduction

One of the most important events in the history of our species has been the development and diffusion of agriculture, which increased greatly the size of the population that could be stably maintained. The introduction of agriculture occurred independently in different periods and in distinct areas of the globe. Concerning Western Eurasia, there is substantial archaeological evidence that agriculture was initially introduced in the Middle East about 10,000 years ago, at the beginning of the Neolithic period, and then spread through the European continent with an estimated rate of about 1 (0.6–1.3) km/yr [1].

An important unresolved question is how this diffusion took place. Two principal models have been proposed: a model in which the population with the technology expands into areas determining a substantial gene flow into the original populations; the demic diffusion model, and a cultural model in which primarily only the information moves into new populations, allowing them to expand. The nature of this diffusion (mostly demographic or cultural) is debated and, like many events of the past, difficult to be unequivocally and rigorously assessed. The suggestion that it was largely demic derives from the first principal component of a map of Europe plotted using geographic location and gene frequencies of a large number of classical pre-molecular markers [2], [3].

More recently, this model has been invoked to explain clinal differences in the distribution of single nucleotide polymorphisms (SNPs) located in the non-recombining region of the Y chromosome (NRY) and in the mitochondrial DNA (mtDNA). Given that the timing of events is crucial to reconstructing the past demography, these analyses considered also another class of genetic polymorphism with a much more rapid mutation rate, the short tandem repeats (STRs) polymorphisms, in which the number of repeated sequences at a locus frequently increases or decreases over the course of generations and thus allows distinctions among members of the same haplogroup. Using these analyses, supporters of the demic diffusion model have proposed that families of lineages defined by certain combinations of SNPs, also known as haplogroups (namely E-M35, J-M172, F-M89 and G-M201 for the NRY and J, and T for mtDNA), represent tracers of the diffusion of farmers from the Middle East during the Neolithic [4], [5]. However, most of the subsequent analyses of the NRY highlighted a much more complex scenario than that originally envisaged by the same authors, who then restricted the set of putative tracers of the demic diffusion from the Middle East during the Neolithic period [4], [5] to only specific subclades (E-78; E-M123; J-M172 and its branches defined by the M67 and M102 mutations [6]). Still, it has also been suggested that E-M78 is not a reliable marker of Neolithic diffusion but instead traced a late Mesolithic spread of people from the southern Balkans towards South-East [7]. Furthermore, some of the variants used for these analyses are relatively frequent and, as illustrated by Currat and Excoffier [8], an ascertainment bias can occur when common variants, which usually are also older, are selected for these analyses. This view is consistent with the fact that such gradients are not observed for mtDNA when unascertained complete sequence data are used [9].

To overcome this, it was also proposed that the farmers migrating into areas occupied by hunter-gatherers were predominantly males [10], [11]. However, while this model is plausible in a limited area and over a limited period of time (for instance this is observed in areas of central Africa where hunter-gatherers still live near farmers) it is difficult to envision a simplified multigenerational scenario where only male farmers repeatedly migrate and spread their genes across Europe over several thousand years.

As a result of increased knowledge and improved genetic resolution the original demic model has been progressively refined and eventually replaced with an hybrid model with both proposed ways of diffusion of agriculture contributing.

Still, the main uncertainty about the early peopling events in Europe is related to the fact that the clines in frequency of genetic variants do not *per se* reveal the time when the migration events underlying them actually occurred and that even the definition of the clines is prone to a number of variables that can affect results and conclusions.

For instance the R- M269 haplogroup shows the highest frequency in Western Europe reaching frequencies as high as 85% in Ireland [12], but it is also very common in the Iberian peninsula, Sardinia and Anatolia [13], [14]. It has been proposed that R-M269 was initially introduced in Europe during the first Upper Paleolithic period and then expanded across the continent after the Last Glacial Maximum [4], [15]. Crucial to this analysis was the observation that by genotyping an adequately informative set of STRs, a specific R- M269 STR haplotype, known as the Atlantic Modal Haplotype may be distinguished. This haplotype is extremely common in the Basque, Welsh, and Irish populations [16] but very rare in Anatolia where another common STR-haplotype is detected in the M269 lineage. From these data there is no evidence, therefore, that at least in the British Isles, the agriculture transition was accompanied by a genetic flow due to incoming Neolithics or later immigrants originating in the Near East [16].

In contrast, Balaresque and colleagues [17] stated that M269-derived Y chromosomes belong to a relatively young group of Y-chromosomes that were distributed over Europe by a process of demic diffusion associated with the spread of farming out of the Middle East, via Anatolia and that demic diffusion associated with the spread of farming out of the Middle East, via Anatolia.

**Figure 1:**
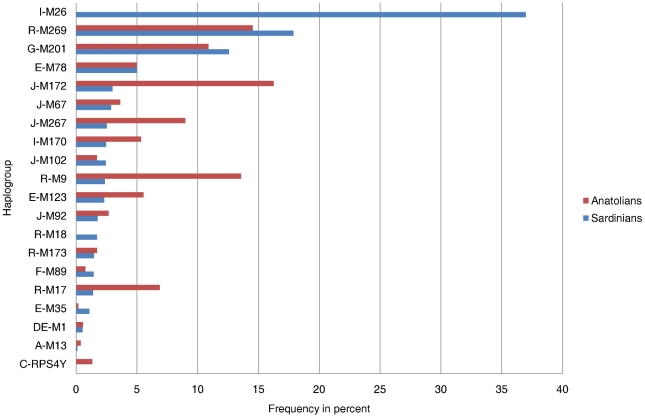
Comparison of haplogroup frequency percentages of the Sardinian and Anatolian Y-chromosomes. Anatolian population data are from [14].

Given these conflicting results, we reasoned that a more genetically informative comparative analysis of the Y-chromosome structure of R-M269 and of the other putative Neolithic tracers in Anatolia and in the rest of Europe, in particular in the island population of Sardinia, would be most revealing. Sardinia is especially important because agriculture arrived later and there is archaeological, genetic [13], [18], [19] and paleontological [20] evidence of pre-Neolithic human occupation, so it could provide more detailed clues to distinguish between the demic diffusion and the cultural spread models. Furthermore, previous analyses showed that, even if Sardinians and Anatolians have a different pattern of distribution for Y-chromosome haplogroups (figure 1), some additional lineages which have been proposed to mark the Neolithic diffusion of farmers are relatively common in this island so a comparison of the haplogroups of Anatolia and Sardinia has important ramifications [13].

## Results

With the aim of comparing the Y chromosome variability of Anatolia [14] with that of Sardinia, we analyzed the STR loci structure of paradigmatic haplogroups selected from previous work [13], [14] on the basis of their frequencies in the assessed populations (figure 1).

The STR loci typed were matched with those previously assessed in Anatolia: DYS19, DYS388, DYS390, DYS391, DYS392, DYS393, DYS389-I, DYS389-II, DYS439, DYSA7.2 [14]. Regarding the haplogroups examined, we initially focused on R-M269 which represents the individual haplogroup most shared not only in Sardinia and Anatolia, but also in Europe as a whole. We then analysed G-M201, which is also common in Anatolia and Sardinia, as well as the putative markers of Neolithic diffusion, E-M78; E-123; J-M172 common in Anatolia but detected at appreciable frequency also in Sardinia (figure 1).

**Figure 2:**
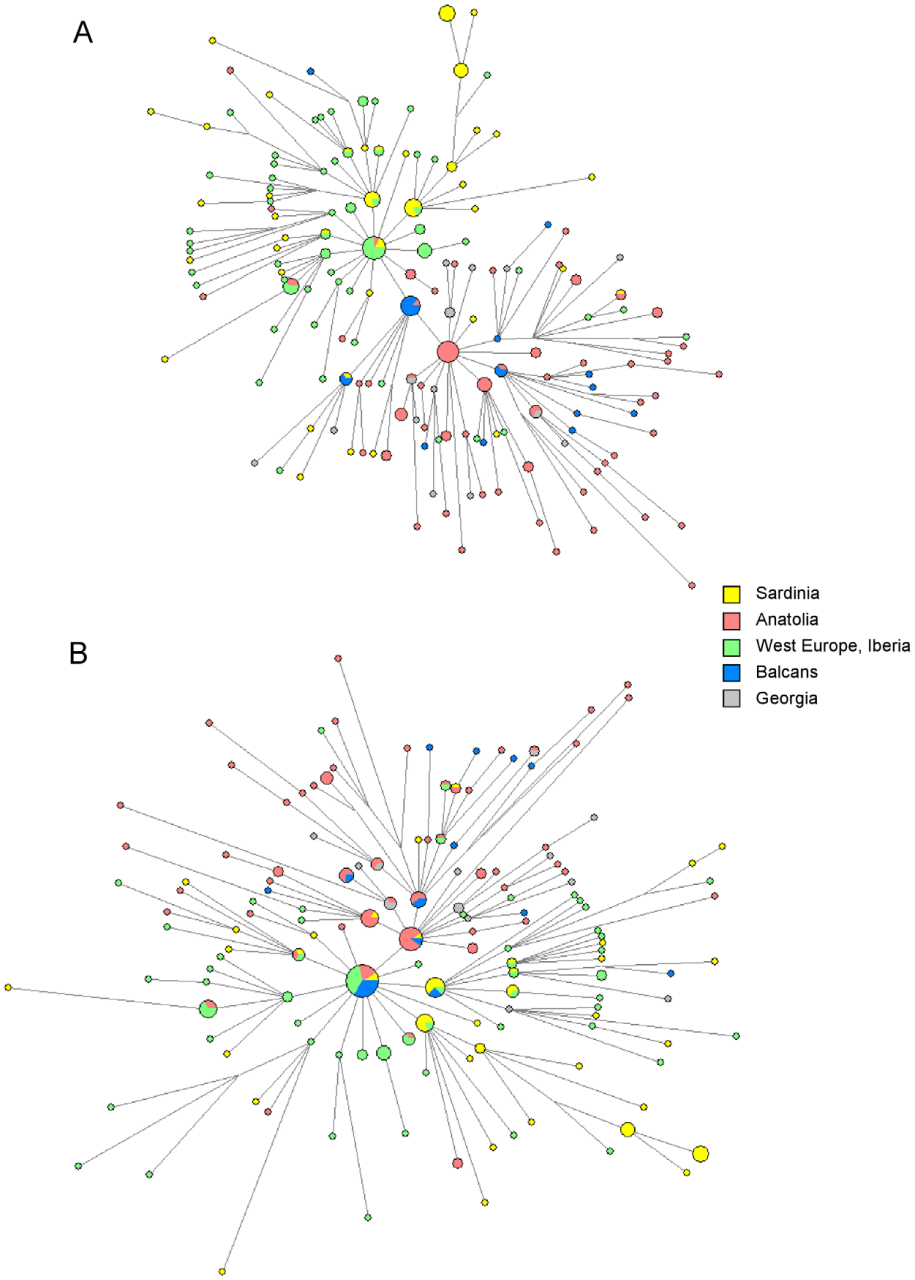
Median-joining network analysis of R-M269 haplogroup lineages. Figure 2A: The entire 10 STR data were used. Figure 2B: The same data of 2A but excluding STR DYSA7.2. The two network comparison highlights the impact on the network topology of the number of STRs used and their informativity. (Data from Anatolian, Georgian, Balkan, North West European and Iberian populations are from [14].

**Figure 3:**
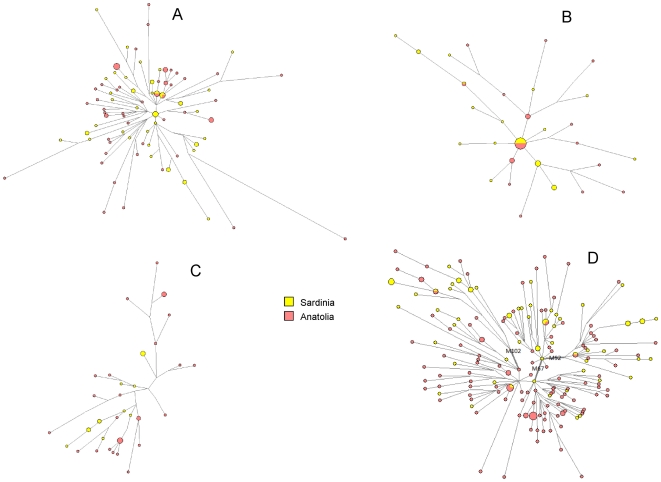
Median-joining network analysis of haplogroup lineages common in Sardinia. A. G-M201; B. E-M78; C. E-M123; D. J-M172 and its subclades. Anatolian population data are from [14].

**Figure 4:**
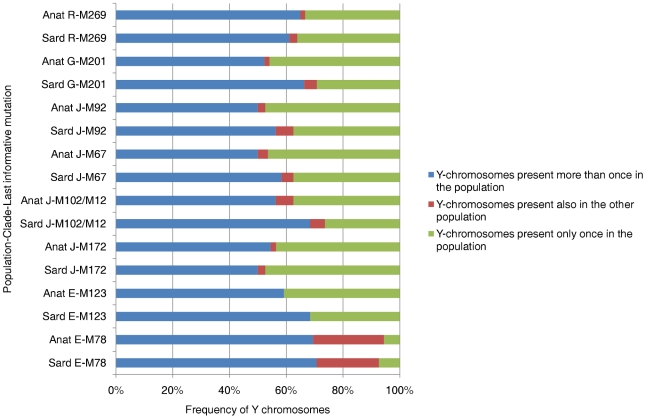
Percentage partition of Sardinian and Anatolian Y-chromosomes in copies shared with the other population, shared within the population and not shared, i.e. present only once considering both populations. Anatolian population data are from [14]. Abbreviations: Anat  =  Anatolian sample, Sard =  Sardinian sample.

For each haplogroup, we analyzed the relevant subclades and constructed networks of their STR haplotypes (shown in figures 2 and 3), estimated Time from the Most Recent Common Ancestor (TMRCA) values (table 1) and performed a detailed analysis of the intra-lineage haplotype sharing in the assessed populations (tables 2–3, figure 4). To promote reliable analysis, and to minimize sampling components of variance, we wanted to ensure that a similar number of chromosomes were counted in Anatolia and in Sardinia for each of the assessed lineages. This was achieved by assessing a subset of 238 Sardinian individuals (table S1) selected from a previous work [13].

**Table 1:** TMRCA values of the main Sardinian and Anatolian haplogroups provided by BATWING analysis using ten STR loci.

	Lineage	I-M26	R-M269	G-M201	E-M78
**Sardinia**	**TMRCA**	17.8	27.0	23.7	13.6
	**95% c.i.**	16.4–29.2	19.5–67.5	23.7–31.8	11.8–21.2
**Anatolia**	**TMRCA**		19.6	22.8	28.6
	**95% c.i.**		19.4–44.4	20.6–32.8	17.9–33.2
**Both populations**	**TMRCA**		32.6	25.8	28.2
	**95% c.i**		25.0–80.7	14.0–37.8	20.9–33.2

**Time is expressed in KYA. Anatolian population data are from** **[14]** **.**

**Table 2:** Number and percentage of 10 STR loci haplotypes shared in Anatolia and Sardinia in representative lineages.

Haplogroup	E-M78	E-M123	J-M172	J-M102	J- M92	J-M67	G-M201	R- M269
N. of entirely genotyped Sardinian samples	29	13	19	13	14	18	59	66
N. of copies present more than once in the Sardinian sample	17	7	0	7	4	4	29	24
% of copies present more than once in the Sardinian sample	58.62	53.85	0.00	53.85	28.57	22.22	49.15	36.
N. of Sardinians copies shared with Anatolians	9	0	1	1	1	2	4	3
% of Sardinian copies shared with Anatolians	31.03	0.00	5.26	7.69	7.14	11.11	6.78	4.55
N. of entirely genotyped Anatolian samples	25	29	85	9	14	19	57	76
N. of copies present more than once in the Anatolian sample	14	9	14	2	0	0	5	35
% of copies present more than once in the Anatolian sample	56.00	31.03	16.47	22.22	0.00	0.00	8.77	46.05
N. of Anatolian copies shared with Sardinians	9	0	3	1	1	1	2	2
% of Anatolian copies shared with Sardinians	36.00	0.00	3.53	11.11	7.14	5.26	3.51	2.63
**P value** *	**0.57**	**N.A.**	**0.56**	**1**	**1**	**0.60**	**0.68**	**0.66**

Anatolian population data are from [14].

*For each lineage, P values are computed using the Fisher exact test and a 2×2 contingency table considering two variables, place of origin (Sardinia or Anatolia) and sharing of STR haplotypes in the two populations (shared or not shared) and tabulating the data accordingly: a) number of counts of observed STR haplotypes present in Sardinia that are shared with the Anatolians, b) number of counts of STR haplotypes present in Sardinia that are not shared with the Anatolians, c) number of counts of observed STR haplotypes present in Anatolia that are shared with the Sardinians, d) number of counts of observed STR haplotypes present in Anatolia that are not shared with the Sardinians.

N.A =  Not Applicable because of the presence of 0 values in cells.

**Table 3:** Percentage of representative lineages in the Sardinian and Anatolian samples and percentage of shared haplotypes with the compared population.

Haplogroup	% in the Sardinian population	% of Sardinian haplotypes shared with Anatolians	% in the Anatolian population	% of Anatolian haplotypes shared with Sardinians
E-M78	4.98	1.55	4.97	1.79
E-M123	2.31	0.00	5.54	0.00
J-M172	3.00	0.16	16.25	0.57
J-M102	2.44	0.19	1.72	0.19
J- M92	1.78	0.13	2.68	0.19
J-M67	2.89	0.32	3.63	0.19
G-M201	12.58	0.85	10.90	0.38
R- M269	17.88	0.81	14.53	0.38
**Total**	**47.86**	**4.01**	**60.23**	**3.69**

Anatolian population data are from [14].

Individually, R-M269 represents the most informative lineage for which previous studies have reached conflicting conclusions. Therefore, to have a more definitive picture we constructed a network of R-M269 considering not only Anatolia and Sardinia but also the Balkan, Georgian, Iberian and North-Western European populations (figure 2A).

A dichotomy between Western and Eastern populations was apparent with two distinct core haplotypes, corresponding to two informative R-M269 STR patterns. On the one hand is the DYS393-13/DYSA7.2-12 STR pattern common throughout Western Europe and the Iberian peninsula, the Atlantic Modal Haplotype [16]. On the other hand is the DYS393-12/DYSA7.2-11 STR pattern which appears as a more recent Eastern European haplotype.

The Sardinian haplotypes belong to the Atlantic Modal Haplotype variability, with an interesting internal differentiation shown by the completely Sardinian branch off-shoot (figure 2A). In contrast, the majority of Anatolian samples belong to the DYS393-12/DYSA7.2-11 subtype. Interestingly, the bridge between the two main forms, is represented by the intermediate step of a haplotype common in the Balkan region, DYS393-13/DYSA7.2-11. This dichotomy is further corroborated when TMRCA values for R-M269 are examined; they provided a value of 32.6 KYA (1000 Years Ago, C.I. 95% 25.0–80.7) in the Iberian sample, 27.0 KYA in the Sardinians, (C.I. 95% 19.5–67.5) and 19.6 KYA (C.I. 95% 19.4–44.4) in the Anatolians: in all cases clearly pre-dating the advent of agriculture.

Quantitative data about a direct comparison between Sardinia and Anatolia are also informative. While the R-M269 haplogroup is the one for which the Sardinians and Anatolians have high and comparable frequencies (17.88% in Sardinia, 14.53% in Anatolia, figure 1, table 3), a more detailed analysis of the intra-lineage STR patterns in these populations revealed important differences. Only 4.55% of the R-M269 Sardinian Y-chromosomes were identical to those examined in Anatolia, while 2.63% of the Anatolian R-M269 were shared with the Sardinian gene pool (figure 4, table 2). The distribution of the counts of the shared and not shared haplotypes in these populations was not significantly different (table 2).

The Sardinian and Anatolian populations also have similar frequencies of another common haplogroup, the G-M201 (figure 1, table 3) having a frequency of 12.6% in the former and 10.9% in the latter populations. The network of the G-M201 haplogroup (figure 3A) showed a “star” topology, but notice that similar frequency sizes are observed in the central core (where only Sardinians are present) and the peripheral haplotypes. Furthermore, some intermediate haplotypes were missing, which might suggest the presence of ancient founder effects or bottlenecks with relevant genetic drift phenomena. Consistently, similarly ancient TMRCA values of 23.7 and 22.8 KYA were found in Sardinia and Anatolia, respectively (table 1). The STR structure of this lineage reveals that 6.78% of the G-M201 haplotypes detected in Sardinia were also detected in Anatolia, while 3.51% of those found in Anatolia were also present in Sardinia (table 2, figure 4). Like R-M269, the distribution of the counts of the shared and not shared haplotypes in these populations was not significantly different (table 2).

We then examined the two main sub-clades of the putative Neolithic E-M35 haplogroup, defined by the M78 and M123 mutations (figure 1). The Sardinian and Anatolian populations had been found to have very similar frequencies of E-M78 (4.98% and 4.97% respectively, figure 1, table 3). We found a “star-like” shape in the network of E-M78 with population-specific clades that departed from a core haplotype shared by the Anatolian and Sardinian populations (figure 3B) and estimated TMRCA values of 13.6 and 28.6 KYA for the Sardinian and Anatolian E-M78, respectively. We have also observed that 31% of the E-M78 Sardinian sub-haplotypes were also present in the Anatolian gene pool, while 36% of the Anatolian E-M78 were present in the Sardinian one (table 2, figure 4). The impressive symmetry of the haplotype sharing (table 2, figure 4), and the evolution pattern inferred by the network (figure 3B) is consistent with a shared equivalent ancestry, followed by subsequent differentiation.

The other putative Neolithic E-M123 mutation was found to be relatively rare in both Anatolia (frequency 5.5%) and Sardinia (2.3%) (figure 1, table 3). Furthermore, none of the E-M123 Y-chromosomes were shared between the two populations (tables 2–3, figure 3C).

Haplogroup J, defined by the 12f2 polymorphism, was found to present a different distribution in Sardinia and Anatolia. In particular, the Sardinian J-M172 and its subclades J-M102, J-M67 and J-M67,92 Y-chromosomes only account for a low percentage of the genetic pool while the Anatolian J-M172(xM102,67) represented 16% of the overall Y chromosomes (figure 1, table 3). Few J haplotypes were shared between the two populations (figures 3D, 4, table 2).

Considering the lineages assessed formerly (E-M78, E-M123, J-M267, J-M172(xM102,M67), J-M102, J-M67(xM92), J-M92, G-M201, I-M26, R-M269) as a whole, we found a small number of STR haplotypes were shared in both populations representing 4.01% of the total number of haplotypes present in Sardinia, and 3.70% of those detected in Anatolia (table 3).

Finally, as shown in Figure 1, one lineage, I-M26, is very common in Sardinia and absent in Anatolia (it is also detected in some Western European populations, albeit rarer than in Sardinia). The distribution of this founder variant, having in Sardinia a TMRCA of 17.8 thousands years (table 1), indicates that it originated before the main initial peopling of Sardinia, but after the separation between the Sardinians and the Anatolians and provides some rough indication about the time of separation between the bulk of these populations.

## Discussion

With the aim of investigating the foundation of the demic diffusion model, we compared the STR loci structure of paradigmatic Y chromosome haplogroups in Sardinia with those observed in Anatolia and in other European populations where they could be detected at appreciable frequencies [14].

R-M269, present at high frequencies in the whole of Europe appears to be singularly the most informative haplogroup. The Sardinians and Anatolians, even if they had very similar R-M269 haplogroup frequencies, could readily be distinguished when informative STRs were considered, with the Sardinians in the Western group, and the Anatolians in the Eastern group. In addition, the two populations belong to two distinct star-like episodes found in the network linking the STR haplotypes carrying the M269 mutation (figure 2A).

This suggested that there have been at least two expansions: one in Anatolia, and another in the Western European regions. The former might have dispersed to Georgia eastward and to the Balkan Peninsula westward, since these populations carried divergent R-M269 haplotypes. The other one could have involved a primitive settlement of R-M269 in Sardinia, considering both the R-M269 STR haplotype's relatedness to the Iberian one, and the TMRCA values with a more ancient date for the Iberian/West European R-M269 clade (KYA 40.7, C.I. 95% 32.6–53.4) followed by Sardinians (KYA 27.0, C.I. 95% 19.5–67.5), Anatolians (KYA 19.6, C.I. 95% 19.4–44.4), and the population of the Balkans (KYA 14.8, C.I. 95% 11.0–16.6) (table 1). This probably means that, today, Western European populations have imprints of a more ancient Upper Palaeolithic peopling while the Eastern populations, like Anatolia, could have later collected in the more recent Upper Palaeolithic age, a different R-M269 modal haplotype.

Aside from the timing difference, the STR content of R-M269 haplotypes also indicates that the Sardinians and other Western populations did not receive this common lineage from settlers coming from the South-East following a demic diffusion model. These results are in agreement with the observations of Wilson and colleagues on the Iberian and British populations [16] and contrast sharply with the data and conclusions presented by Balaresque and collegues [17].

In the latter study, a network analysis of R-M269, revealed a starlike topology and TMRCA values for this haplogroup that were interpreted as consistent with a Neolithic demic expansion. Furthermore, a positive correlation of the haplotypes variance with the longitude was also reported as consistent with the spread of farming out of the Middle East. However, there may be some simple explanations for this apparent discrepancy.

First, the STRs used in our study are more numerous than in the work of Balaresque et al. [17]; specifically we typed also the marker DYSA7.2 (also called DYS461) and they did not. This marker is critical for haplotype identification. In fact, when we repeated our analysis but excluded marker DYSA7.2, the resulting network goes from a markedly bipolar structure (figure 2A) to a starlike one (figure 2B) that strongly resembles that of Balaresque et al. [17]. This STR, along with DYS393, allow the demarcation of the haplotype known as the Atlantic Modal Haplotype [12], [16], [21]–[23] that determines the characteristic bipolar topology of our network R-M269 in the analyses that include this variant.

Second, Balaresque et al. [17] used a STR specific germ-line mutation rate that placed the TMRCA in the Neolithic age. In contrast we used a unique prior for the microsatellite mutation rate estimates as 6.9×10^−4^ as recommended by Zhivotovsky and co-workers [24]–[26], see also [27]–[31], that, as reported above, placed the haplogroups TMRCA values in pre-Neolithic times. The difference between the former, evolutionarily effective, and the latter, germ line mutation rates is critical. In fact the haplogroups that survive the stochastic processes of drift and extinction accumulate STR variation at a lower rate than predicted from corresponding pedigree estimates. In particular, under constant population size, the accumulated variance is on average 3–4 times smaller [26]. Hence germ line mutation rates provide evolutionary estimates for haplogroups biased toward much younger age [26]. Also the correlation between longitude and the variance reported by Balaresque and co-workers [17] is skewed by the obtained TMRCAs values. If the TMRCAs are more ancient, as in our computations based on the evolutionarily effective mutation rate, the progenitor will be located in pre-Neolithic times. The distribution of populations in figure 2A of Balaresque et al. [17] is also compatible with the post-glacial repopulation of areas further north. Furthermore, even the genetic landscape of the South-Eastern populations that acquired the Neolithic technology radiating from Anatolia, seemed to be shaped by autochthonous demographic expansions not related to the spread of people from Anatolia [7]. Overall, these observations indicate that the presence/absence of a single STR marker in the network can critically affect analyses, interpretation of the data and conclusions as does the use of different STR mutation rates.

They also illustrate the risk of a reductionist model focusing only on one individual haplogroup without considering more realistic population dynamics; groups of individuals - not just specific lineages - move from one population to another in the presence of a real gene flow, and the mechanisms of diffusion of cultural knowledge may have also differed over time and geographic area. For instance an incisive way to further assess the demic diffusion model is to use robust quantitative data to compare the intra-lineage variation - defined by STRs–not only of R-M269 but also of the additional putative “Neolithic tracers” in Anatolia and in any other European test population in which they are sufficiently common and where agriculture was introduced later. If these variants are genuine tracers of the demic diffusion, the test population should include these lineages, and when present they should contain a subset of the STR variability present in Anatolia. Some of these markers are present at appreciable frequencies in Sardinia and we therefore used a combination of Y-chromosome SNPs and STRs data and matched data in Anatolia (as a test donor population) and in Sardinia (as a test recipient population) for the presence of genetic flow related to the introduction of agriculture.

We observed that the percentage of intralineage STR-haplotypes shared between Sardinians and Anatolians are consistently very small (figure 4, table 3). Furthermore, the proportion of individuals with STR haplotypes shared in Sardinia and in Anatolia, relative to the proportion of individuals without shared STR haplotypes, was rather similar and do not differ statistically in the two populations for R-M269 and for all assessed haplogroups (figure 4, table 2). These data, along with the high-resolution STR structure and distribution of the various haplogroups and the related TMRCA values also indicate that the shared variability amongst these populations is best explained by an earlier dispersal of these haplogroups from a common ancestral gene pool, and subsequent ancient founder effects covering a long period of time in the pre-Neolithic age.

Hence, also this set of analyses clearly indicates that, at least in Sardinia, the genetic contribution of the Neolithic settlers was negligible, despite the presence of Y-chromosome lineages that have been considered specific markers of such diffusion. Indeed, all together the various sets of data suggest that the clines of frequencies observed in Europe for some other markers predate the introduction of agriculture and that the E, G and J clades also came to Sardinia by a pre-Neolithic pathway. It could be argued that this latter set of analyses are valid for Sardinia; an exception that cannot be generalized to the rest of Europe. However, the fact that lineages, such as G-M201, E-M78, E-M123, J-M172 are rare or absent in Central, Western and Northern Europe, is strong primary evidence against the assertion that these variants are tracers of Neolihic diffusion from the Middle East to the rest of Europe. Furthermore, a similar trend can be seen also for autosomal traits like beta-thalassemia variants that show different patterns of distribution in these populations [32]. If a considerable fraction of Neolithic farmers arrived in Sardinia and elsewhere, the ancient–IVS110 beta-thalassemia mutation (like the Eastern subtype of R-M269) common in Anatolia and in the Middle East, would be detected at appreciable frequencies in these populations, at least where beta-thalassemia is common [32].

We can conclude that our data are not consistent with the hypothesis that there was a significant diffusion of genes into Western Europe driven by the acquisition of agriculture during the Neolithic age and support the notion that knowledge can spread faster than the genes of its discoverers.

## Materials and Methods

### Sample selection

930 male samples genotyped for the biallelic markers M1, M9, M13, M17, M18, M26, M35, M67, M68, M78, M89, M92, M102, M123, M130, M170, M172, M173, M201, M267 and M269 and 585 samples genotyped both for biallelic and for the STR haplotype DYS19, DYS385a, DYS385b, DYS389-I, DYS389-II, DYS390, DYS391, DYS393 all located in the non-recombining portion of the Y chromosome, were available from a previous work [13].

We selected from this DNA collection 238 I-M26, R-M269, J-M172, E-M35, R-M18, G-M201 samples of Sardinian origins (See sample selection of Contu *et al.* [13]) and genotyped the additional DYS392, DYS388, DYS439, DYSA7.2 STR loci.

### DNA typing

The following primers were used to amplify fragments of interest: DYS392: FOR-TCA TTA ATC TAG CTT TTA AAA ACA A; REV-AGA CCC AGT TGA TGC AAT GT. DYS388: FOR-GTG AGT TAG CCG TTT AGC GA; REV-CAG ATC GCA ACC ACT GCG. DYS439: FOR-TCC TGA ATG GTA CTT CCT AGG TTT; REV- GCC TGG CTT GGA ATT CTT TT. DYSA7.2: FOR-AGG CAG AGG ATA GAT GAT ATG GAT; REV- TTC AGG TAA ATC TGT CCA GTA GTG A. Y-STR loci were genotyped by separating the fluorescent-tagged PCR products on a 96-capillary-sequencer (MegaBACE 1000 DNA capillary sequencer) following the manufacturer's instructions. Two samples for each STR locus and two different allelic sizes were sequenced by direct capillary sequencing. The individuals were genotyped based on allele size data. Sequence patterns of DYS388, DYS439 and DYSA7.2 loci were converted to number of repeats following the recommendations of Gusmão et al. [33].

### Nomenclature

The International Society of Genetic Genealogy [34] published a strictly cladistic Y-DNA haplogroup tree based on capital letters, in order to identify the broader clades, and a succession of numbers and letters for lower hierarchical levels, thus flexible enough to allow the unambiguous naming of haplogroups defined by newly discovered downstream markers. However, the internal nodes are highly sensitive to changes in tree topology cause the addition of new SNPs. This occurrence may require the periodical update of the nomenclature and can generate disorder when comparing data between papers published in different times. So, to overcome possible ambiguities and identify a given lineage, we added to the main letter defining the haplogroup name, the last downstream SNP observed. Thus we used E-M35 (E1b1b1), E-M78 (E1b1b1a), E-M123 (E1b1b1c), G-M201 (G), I-M26 (I2a1), J-M267 (J1), J-M172 (J2), J-M67 (J2a4b), J-92 (J2a4b1), J-M102 (J2b), R-M173 (R1), R-M18 (R1b1a1) and R-M269 (R1b1b2) to define lineages in this work.

### Data Analysis

Haplotype sharing analyses were performed using the Arlequin 3.01 package [35].

Haplotype data were used to construct haplotype networks using Network program v5.0.0 and default parameters were used for obtaining the median joining network trees [36].

The program BATWING [37] was used for a genealogical analysis. BATWING uses Markov chain Monte Carlo (MCMC) techniques to sample many reconstructed genealogies proportional to their probability under the coalescent model in a Bayesian framework (for background see Wilson *et al.* [37]). These reconstructed population histories depend on models for mutation and the expected genealogical structure and prior distributions for parameters of interest. By summarizing the population histories we can see the sorts of population history and ranges of parameters that are consistent with the data in the present. At equilibrium, the set of most likely population histories (represented as trees) is obtained by sampling from the posterior probability distribution of all possible trees, given the observed data and the assumed underlying genetic and demographic model. The extended BATWING version used here assumes an unbounded single stepwise mutation model for the microsatellite loci and a coalescent process under an exponential model of population growth from an initially constant-size population.

We used BATWING analysis to establish the individual TMRCA for the I-M26, R-M269, G-M201 and E-M78 haplogroup lineages, using the Sardinian data and those from the other populations. A unique prior for the microsatellite mutation rate was based on the Zhivotovsky et al. [24]–[26] estimate as 6.9×10^−4^ and applied to DYS19, DYS388, DYS389I, DYS389II, DYS390, DYS391, DYS392, DYS393, DYS439 and DYSA7.2, setting gamma as (1.47, 2130) (mean = 0.00069, SD = 0.00057) [38]. In this analysis we avoided using samples containing the duplicated DYS19 microsatellite.

Weakly informative priors were also given other parameters to aid convergence of the MCMC process as described in the Contu *et al*. [13]. Generation time was set at 25 years as used elsewhere in Y-chromosome studies [39]–[42], adapting the estimated generation times for present day males to the presumably shorter life span in the past [43]. Although a natural measure of the central tendency of a sample of continuous data is its mode (the most probable value), the mean and median are the most popular measures of location due to their simplicity and ease of estimation. The median is often used instead of the mean for asymmetric data because it is closer to the mode and is insensitive to extreme values in the sample.

However, since the distribution of our simulated data appeared definitely skewed, non-normal and exposed to unpredictable contamination depending on the Markov Chain Monte Carlo sampling of the state space, after verifying that none of the markers examined showed bi-modal distribution, we employed the Half-Range Mode [44]. This is based on subsequent subdivision of the data set in an iterative fashion and appears to be one of the best compromises between reliability, ease of implementation and computing time. Indeed Half-Range Mode is robust for a wide variety of distribution and contamination levels [45]. This mode estimate also seems less affected by the sample size effect observed with mean-based estimates since, in our data, good convergence for the mode could be obtained even with relatively small sampling (∼10^7^), resulting in a good compromise between accuracy of calculation and computation time.

The inferred BATWING values are conservative, since we used a generation time of 25 years and the more robust and reliable mode-based estimates instead of the more commonly-used mean and median measures that tend to provide much older values for all the parameters assessed with BATWING [13], [44], [45].

Finally, we have employed a step-wise analytical strategy addressed to initially test for the presence of genetic flow from the Middle East (and notably from Anatolia) to any another European test population (and notably to Sardinia) and then, if a genetic flow was detected, to assess the size of this flow by incorporating suitable statistical models into the analyses.

To assess for the presence of detectable genetic flow from Anatolia we have used both qualitative data (the network analysis described above) and quantitative data to test the following hypotheses. If any lineage is indeed a genuine tracer of Neolithic diffusion from Anatolia, 1) it must be present in the recipient populations and 2) when present its intra-haplotype variation defined by differences in length of the STRs should be a proper subset of that observed in Anatolia. To test this, for each lineage, *P* values were computed with a 2×2 contingency table using the Fisher exact test. More specifically, for each haplogroup present in both populations, we partitioned the data into mutually exclusive subsets based on their distribution in the assessed populations. We compared a) number of observed STR haplotypes present in Sardinia that are shared with the Anatolians, b) number of observed STR haplotypes present in Sardinia that are not shared with the Anatolians vs c) number of observed STR haplotypes present in Anatolia that are shared with the Sardinians, d) number of observed STR haplotypes present in Anatolia that are not shared with the Sardinians. If the intra-lineage variation doesn't show statistically different proportions in the two populations, it would reject the hypothesis of a detectable unidirectional gene flow from Anatolia, while on the other hand, if these counts are statistically different in the two populations with the higher proportion of shared haplotypes in Sardinia (and in any donor test population) hence indicating that its variability is encompassed in the Anatolian variability, it would suggest an unidirectional gene flow from the East.

The size effect of any genetic flow, if observed, could be further assessed by simulating data under different models and comparing simulated data summaries with the observed data. This more complex analysis would serve to reconstruct the separate contributions of complex migratory waves, and to take into account parameters, such as the initial effective population size, the degree of admixture with local population, the effect of gene flow with neighboring population over time as well as of convergent evolution at STR loci.

## Supporting Information

Table S1 Genotyping data.
(0.10 MB PDF)
