# Supplementary material for: A Comparison of Y-Chromosome Variation in Sardinia and Anatolia Is More Consistent with Cultural Rather than Demic Diffusion of Agriculture
Source: PLoS One. 2010 Apr 29;5(4):e10419. doi: 10.1371/journal.pone.0010419 (PMC2861676; doi:10.1371/journal.pone.0010419)
Supplement: Table S1 — Genotyping data. (0.10 MB PDF) [file pone.0010419.s001.pdf]

Supplementary data

Id, Freq.,S19,S390,S391,S392,S393,S389a,S389b,S388,S439,SA72

R-M269

CA1	2	14	24	11	13	13	14	16	12	13	12
CA111	4	14	24	10	13	13	13	16	12	12	12
CA226	5	14	24	11	13	13	13	16	12	13	12
CA236	1	14	23	11	14	13	15	16	12	13	12
CA244	1	14	23	11	14	13	13	16	12	13	12
CA252	1	14	25	10	13	12	13	16	12	12	12
CA266	1	14	24	10	13	12	14	16	12	13	12
CA278	1	14	21	11	13	13	13	16	12	11	12
CA36	5	14	23	10	13	13	13	16	12	12	12
CA4	4	14	23	11	14	13	14	16	12	13	12
CA49	1	14	24	10	13	13	13	17	12	12	11
CA63	1	14	24	10	13	14	13	16	12	11	11
CA92	1	15	23	10	14	13	13	16	12	11	13
DIA23	2	14	24	11	13	13	13	16	12	12	12
DIA33	1	14	24	10	13	13	13	16	12	11	12
DIA34	1	14	25	11	13	14	13	16	12	11	11
DIA37	1	14	23	11	14	13	14	17	12	13	12
DIA39	1	14	24	10	13	13	13	16	12	12	11
DIA40	1	14	23	10	13	14	14	16	12	12	12
DIA49	1	14	24	11	14	13	13	18	12	13	13
DIA55	1	15	23	11	13	13	13	16	12	12	12
DIA59	1	14	24	12	13	13	13	16	12	13	13
DIA66	1	14	24	11	13	13	13	16	12	11	12
DIA74	1	14	24	10	14	13	14	16	12	12	12
SOR41	1	14	23	10	15	13	14	16	12	13	12
SOR46	1	14	25	11	13	13	14	16	12	13	12
SOR87	1	14	24	11	13	12	13	16	12	12	10
SOR92	1	14	24	11	13	12	13	16	12	13	10
SOR98	1	14	23	10	14	13	14	16	12	13	12
SOR99	1	15	24	10	13	13	13	16	12	11	13
TEM105	1	14	24	10	13	13	13	17	12	12	12
TEM108	1	14	24	11	13	13	12	16	12	11	12
TEM186	1	15	24	11	13	15	14	16	12	11	12
TEM187	1	15	24	11	13	13	14	16	12	13	11
TEM199	1	14	24	11	14	13	13	16	12	12	12
TEM305	1	14	24	11	13	13	13	16	14	12	12
TEM69	1	14	24	11	13	14	14	15	12	13	12
TEM85	1	14	24	11	13	13	13	17	12	11	12
TEM96	1	14	24	11	13	14	14	16	12	12	12

G-M201

CT29	1	15	22	10	11	13	13	17	12	13	12
DIA137	2	15	23	10	12	14	12	17	12	11	11
CT71	1	15	23	11	11	13	12	17	12	12	11
SOR100	2	15	23	10	11	14	12	17	12	11	11
CT99	1	15	22	10	11	14	12	16	12	11	11
DIA232	2	15	22	11	11	13	14	17	12	13	12
DIA15	2	15	23	10	11	14	12	16	12	11	11
CT156	1	16	24	10	11	14	14	17	12	12	11
CT165	1	15	21	10	11	13	12	16	12	12	11
CT205	1	14	22	10	11	14	12	16	12	12	12
CT207	1	15	21	11	11	14	12	17	12	11	12
CT226	1	15	22	9	11	14	12	16	12	11	9
CA42	2	15	22	10	11	13	14	16	12	12	12
DIA77	4	15	22	10	11	14	12	17	12	12	11
DIA178	2	15	22	11	11	14	13	17	12	11	12
SOR94	2	15	22	11	11	13	13	16	12	12	12
CA57	1	15	23	10	11	14	11	17	15	12	11
CA67	1	15	23	10	11	14	12	17	12	10	12
SOR28	3	15	21	10	11	14	12	17	12	11	11
CA78	1	16	23	10	12	14	12	17	12	12	11
CA85	1	15	23	10	11	13	12	15	12	11	13
CA232	1	15	22	10	11	14	13	17	12	12	12
SOR62	1	15	21	10	11	14	12	17	12	13	12
SOR71	1	16	22	11	11	14	13	17	12	12	12
SOR76	2	15	23	10	11	13	12	17	12	11	11
SOR77	1	15	22	10	12	13	14	16	12	12	12
TEM316	1	15	22	10	11	13	13	17	13	11	11
DIA32	1	15	22	11	11	14	12	16	12	12	11
DIA60	1	15	22	11	11	13	14	16	12	13	12
DIA65	1	15	24	10	11	14	12	18	12	11	11
DIA83	1	15	22	10	11	14	13	17	14	11	11
DIA101	1	15	22	10	12	14	12	18	12	13	11
DIA167	1	16	21	10	11	14	12	16	12	12	11
DIA174	1	15	22	11	11	14	12	17	12	12	11
DIA217	1	16	25	10	11	13	14	17	12	12	12
DIA233	1	15	23	11	11	15	12	17	12	11	11

E-M78

CT77	1	15	23	10	11	14	14	17	12	12	12
CA112	1	14	25	10	11	13	13	17	12	12	12
CT198	1	14	24	10	11	13	13	19	12	12	12
DIA203	1	14	24	10	11	13	13	17	12	11	12
CA228	1	13	24	10	11	13	14	16	12	12	12
CA97	1	13	24	10	11	13	13	17	12	11	12

DIA152	4	13	24	10	11	13	13	17	12	13	12
DIA2	1	13	24	10	11	12	13	17	12	12	12
DIA230	1	13	25	10	11	13	13	17	12	12	12
DIA262	1	13	24	10	11	13	13	18	12	11	12
DIA35	1	13	24	10	10	13	12	19	12	10	12
DIA374	2	13	24	10	10	13	12	18	12	10	12
DIA378	8	13	24	10	11	13	13	17	12	12	12
DIA448	3	13	24	10	11	13	13	17	13	13	12
TEM232	1	13	24	10	11	13	12	17	12	10	12
TEM282	1	13	25	10	11	13	13	19	12	12	13

E-M123

T106	1	13	25	10	11	13	13	18	12	11	11
CT6	3	13	24	10	11	12	13	17	12	10	11
CT88	1	13	24	10	11	13	12	18	12	12	12
DIA118	1	13	23	10	11	13	12	17	12	12	13
DIA147	1	14	25	10	11	13	13	18	12	11	11
DIA422	2	13	25	10	11	13	13	19	12	13	13
DIA429	1	13	24	10	11	13	13	18	12	12	13
SOR5	1	13	25	11	11	13	13	20	12	13	13
TEM169	2	13	25	10	11	13	13	19	12	12	13

J-M172(xM67, M92)

CT11	1	14	25	10	11	12	13	17	14	12	13
CT31	1	14	24	10	11	13	13	16	15	13	12
CT66	1	15	23	10	11	12	13	16	15	12	12
CT73	1	14	25	10	11	12	13	16	15	12	13
CT100	1	14	23	10	11	12	14	17	16	12	13
CT189	1	15	25	10	11	13	13	16	14	12	12
DIA4	1	15	23	9	11	12	13	16	17	12	13
DIA112	1	15	23	9	11	12	13	16	16	13	13
DIA193	1	14	25	10	11	12	13	16	15	11	13
DIA205	1	14	23	10	11	12	14	16	15	11	12
DIA236	1	14	24	10	11	12	14	16	15	11	12
DIA294	1	15	23	10	11	12	13	17	14	12	11
DIA341	1	17	23	10	11	13	14	15	12	11	11
DIA350	1	14	24	10	11	12	13	17	14	12	12
DIA354	1	15	25	10	11	14	13	16	14	13	12
DIA406	1	14	23	10	11	12	13	17	14	12	11
DIA461	1	15	25	10	11	13	13	16	14	13	12
DIA477	1	14	23	10	11	12	13	16	15	11	13

J-M67

CT39	1	14	22	10	11	12	13	17	15	12	11

CT96	1	14	23	10	11	12	13	18	15	11	13
CT112	2	14	23	10	11	12	13	18	15	10	12
CA286	1	14	24	10	11	12	13	17	15	11	13
TEM49	1	14	23	10	11	12	13	18	15	12	13
TEM234	1	15	23	9	11	12	13	16	14	13	13
DIA63	1	14	23	10	11	12	13	18	15	10	13
DIA153	1	14	23	10	11	12	14	16	15	11	12
DIA176	1	16	23	10	11	12	14	16	15	11	13
DIA255	1	16	23	10	11	12	14	16	15	10	13
DIA289	1	14	23	10	11	13	13	17	15	10	12
DIA310	1	14	23	10	11	12	14	17	15	12	13
DIA355	2	15	23	10	11	12	13	16	15	11	13
DIA379	2	14	23	10	11	12	14	17	15	11	13
DIA451	1	14	23	10	11	12	13	16	15	11	13
J-M92											
DIA450	1	14	22	10	11	12	12	16	15	11	13
DIA38	2	15	22	10	12	13	13	17	15	12	12
DIA436	2	15	22	10	11	13	13	17	15	12	12
DIA48	1	14	22	10	11	12	13	16	15	11	13
DIA368	1	13	24	10	11	13	13	17	15	12	12
CA61	1	15	22	10	11	13	13	16	15	12	12
CA86	1	15	22	10	12	13	13	16	15	12	12
CT206	1	14	23	10	11	14	13	17	15	12	13
DIA284	1	14	22	10	11	12	14	16	15	11	13
CA45	1	15	23	10	11	13	13	14	15	12	12
CT158	1	14	22	10	11	12	13	16	13	11	13
DIA290	1	15	22	10	12	13	13	17	16	12	12
I-M26											
CT55	4	16	23	10	11	13	13	15	13	11	11
CT132	2	17	23	10	11	13	13	15	13	12	11
CT22	1	17	23	10	11	12	13	15	13	12	12
CT102	2	16	23	10	11	13	13	17	13	12	11
CT111	2	16	24	10	11	13	13	15	13	10	11
CT109	3	17	23	10	11	13	13	15	13	11	11
CT46	1	18	23	10	11	13	12	15	14	12	11
CT54	1	16	23	11	12	13	13	15	13	12	11
CT85	1	17	23	11	11	13	13	15	13	11	11
CT93	1	15	23	10	11	13	13	15	13	11	11
CT105	1	15	23	11	11	12	13	16	13	12	11
CT114	1	15	22	10	11	13	13	17	13	12	12
CT115	1	16	23	10	11	12	14	15	13	12	11
CT122	1	16	24	10	11	13	13	14	13	10	11
CT126	1	16	23	10	11	13	14	17	13	11	12

CT127	1	17	23	10	11	13	13	16	13	11	11
CT131	1	17	24	10	11	13	14	15	13	11	11
CT171	1	15	23	10	11	13	13	14	13	11	11
CT172	1	17	24	10	11	13	13	15	13	10	11
CT177	1	14	24	11	13	13	13	16	12	12	13
SOR40	1	16	23	10	11	13	13	15	15	10	12

R-M18

CA269	11	15	24	10	13	13	13	15	12	12	12
DIA97	1	15	22	10	13	13	13	15	12	12	12
DIA426	1	15	24	11	13	13	14	15	12	12	12
